# Serological Assessment of *Lyme borreliosis* in Bulgaria: A Nationwide Study

**DOI:** 10.3390/pathogens13090754

**Published:** 2024-09-02

**Authors:** Kim Ngoc, Iva Trifonova, Teodora Gladnishka, Evgenia Taseva, Elitsa Panayotova, Iva Vladimirova, Vladislava Ivanova, Eleonora Kuteva, Iva Christova

**Affiliations:** Department of Microbiology, National Center of Infectious and Parasitic Diseases, 1504 Sofia, Bulgaria

**Keywords:** *Lyme borreliosis*, seroprevalence, *Borrelia burgdorferi*

## Abstract

*Lyme borreliosis* (LB), a tick-borne infection caused by bacteria in the *Borrelia burgdorferi* sensu lato complex, is increasingly prevalent on the Balkan Peninsula, including Bulgaria, where it is the most common tick-borne disease. This study aimed to assess the seroprevalence of LB across Bulgaria by analyzing 1892 serum samples for specific IgG antibodies using a two-tier testing protocol involving an ELISA and immunoblot methods. The results revealed an overall seroprevalence rate of 5.4%, with significant variation based on age, sex, and residence. Seroprevalence increased with age, peaking at 8.4% in individuals over 65 years. Males had a seroprevalence of 8.4% compared to 3.3% in females, and rural residents showed higher seroprevalence (10.2%) compared to urban residents (4.4%). Regional analysis indicated that seroprevalence ranged from 0.0% to 20.0%, with higher rates in northern provinces such as Gabrovo (18.9%) and Targovishte (20.0%). This study highlights the importance of two-step testing protocols for accurate diagnosis and underscores the need for increased awareness and further research to enhance public health measures and the management of LB in Bulgaria.

## 1. Introduction

*Lyme borreliosis* (LB) is a vector-borne infection caused by bacteria in the *Borrelia burgdorferi* sensu lato (*Bbsl*) complex and is transmitted to humans through the bite of infected Ixodid ticks. It is the most prevalent tick-borne infection in Europe and is considered an emerging disease due to its increasing incidence and geographic expansion over the past few decades [1,2,3]. In the early stage, patients with LB may present with a pathognomonic skin lesion and erythema migrans, but some may be asymptomatic or have non-specific flu-like symptoms. After systemic dissemination, the disease can affect the skin, nervous, musculoskeletal, and cardiovascular systems and, if left untreated, has the potential to cause severe long-term complications with a significantly reduced quality of life for individuals [1,2,3,4,5,6].

In Bulgaria, LB is the most common tick-borne disease with a mean annual incidence over the past 5 years of 3.64 per 100,000 of the population [7,8]. This is most likely an underestimation of the true incidence due to various factors such as underreporting, asymptomatic infections, misdiagnosis, limited awareness, and false-negative results during the early stages of the disease. Epidemiological risk factors are related to tick population density and infection rates, presence of reservoir hosts, and human participation in outdoor activities for occupational and/or recreational purposes [9].

Studies on Ixodid ticks in Bulgaria have revealed an infectivity rate of 31–49% among adult ticks in Sofia and Pleven provinces [10,11] in contrast to the low tick infection rate (1.7%) reported in southeastern Bulgaria [12]. Serologic reactivity to *Bbsl* has been found in various animal hosts, including wild birds [13], horses [14,15], dogs, sheep, and cows [16,17], and *Bbsl* have been detected by PCR in rodents [18,19]. However, there are limited data on the seroprevalence of LB among the human population in Bulgaria. Serological studies from neighboring countries report seropositivity rates ranging between 2.3% and 16.2% [20,21,22,23,24,25,26]. In order to better assess the true disease burden and identify high-risk areas for LB, a broader serological investigation among the Bulgarian population is needed.

The aim of this study was to determine the seroprevalence rate of LB among the Bulgarian population by testing serum samples from individuals from all provinces of the country for the presence of specific IgG antibodies against *Bbsl*. In addition to contributing to the ongoing surveillance efforts for LB in Europe and particularly on the Balkan Peninsula, these results will help guide public health planning and increase awareness among clinicians and the general population.

## 2. Materials and Methods

Serum samples (n = 1892) were collected in December 2023. They were randomly enrolled from healthy individuals from all 28 provinces of the country who visited the laboratories for routine biochemical and preventive examinations. Only adults over 18 years of age were included. Information regarding the sex, age, and place of residence (rural or urban) of the participants was recorded.

A standard two-tier testing protocol was applied; samples were initially screened using an ELISA method, and borderline and positive samples were subsequently confirmed with an immunoblot test. Both tests were performed with commercially available kits: Anti-*Borrelia* plus VlsE ELISA (IgG), Euroimmun, Medizinische Labordiagnostika (Lübeck, Germany), EI 2132-9601-2 G, and Anti-*Borrelia* EUROLINE-RN-AT (IgG), DN 2131-3201 G according to the manufacturer’s instructions. The ELISA results were calculated semiquantitatively as the ratio between the extinction of the test sample and that of the calibrator. The interpretation of the results was as follows: positive if the ratio was ≥1.1 and negative if the ratio was <1.1. A ratio ≥ 0.8 but <1.1 was interpreted as borderline. Immunoblot strips included the following specific antigens: VlsE Ba, VlsE Bb, VlsE Bg, p18, p19, p20, p21, p58, OspC (p25), p39, p83, Lipid Bb, and Lipid Ba. Incubated test strips were evaluated with EUROLineScan Software v. 3.4.37 (YG 0006-0101, Euroimmun). The presence of any of the VlsE bands or at least two positive bands is indicative of a positive result.

Further analyses of the results were conducted based on participants’ age, sex, province, and place of residence. The statistical analyses were performed with IBM SPSS Statistics v.26. Pearson’s Chi-square test, multiple logistic regression, and odds ratios (ORs) were used to estimate the risk factors and associations with seropositivity. A *p*-value <0.05 was considered statistically significant.

Ethical approval for this study was obtained from the institutional review board at the National Center of Infectious and Parasitic Diseases (NCIPD) (approval number 5/17.10.2023).

## 3. Results

### 3.1. Lyme borreliosis Seroprevalence in the General Bulgarian Population

A total of 1892 serum samples were tested for the presence of anti-*Borrelia*-specific IgG antibodies. The mean age of the participants was 54.79 years (SD ± 17.20); 778 males and 1114 females were included, and 1578 were from urban and 314 from rural areas. For analysis of the obtained results, the participants were divided into three age groups: 18–39, 40–64, and over 65 years of age.

**Figure 1:**
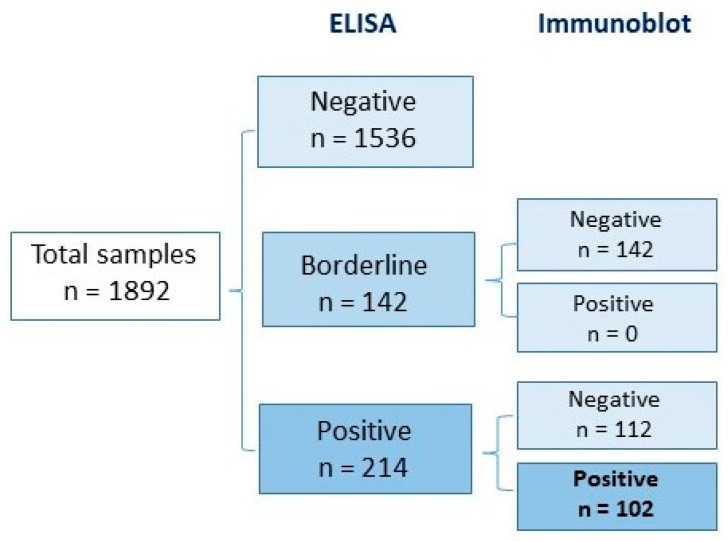
Seropositive samples tested for specific anti-*Borrelia* IgG antibodies with ELISA and immunoblot methods.

The presence of specific IgG antibodies was detected in 18.8% (356/1892) of the tested samples using the ELISA method. Of these, 11.3% (214/1892) were positive and 7.5% (142/1892) were borderline. All positive and borderline results were further tested using the immunoblot method, and 28.7% (102/356) were confirmed to be positive. Overall, the seroprevalence in the studied population was 5.4% (102/1892) (Figure 1).

Seropositivity increased with age, from 2.7% in the 18–39 age group to 4.5% in the 40–65 age group and 8.4% in participants over 65 years of age. Statistical significance was established between age groups 18–39 and over 65 (*p* < 0.0001) and between age groups 40–65 and over 65 (*p* = 0.0005). The ORs were as follows: 0.59 (95% CI 0.30–1.15) for 18–39/40–65, 0.51 (95%CI 0.34–0.78) for 18–39/over 65, and 0.30 (95% CI 0.16–0.59) for 40–64/over 65.

We found a statistically significant difference (*p* < 0.0001) in prevalence between males (8.4%) and females (3.3%). The OR was 2.64 (95% CI 1.75–3.98).

The seroprevalence rate in participants living in rural areas was about 2.5 times higher than in those living in urban areas (10.2% vs 4.4%, *p* < 0.05). The OR was 0.44 (95% CI 0.28–0.67). The results are presented in Table 1.

The multivariable regression analysis found statistical significance for all variables of interest: age, sex, and rural or urban residence (data is presented in Table S1 in the Supplementary Materials). 

### 3.2. Geographic Distribution of Lyme borreliosis Prevalence

**Figure 2:**
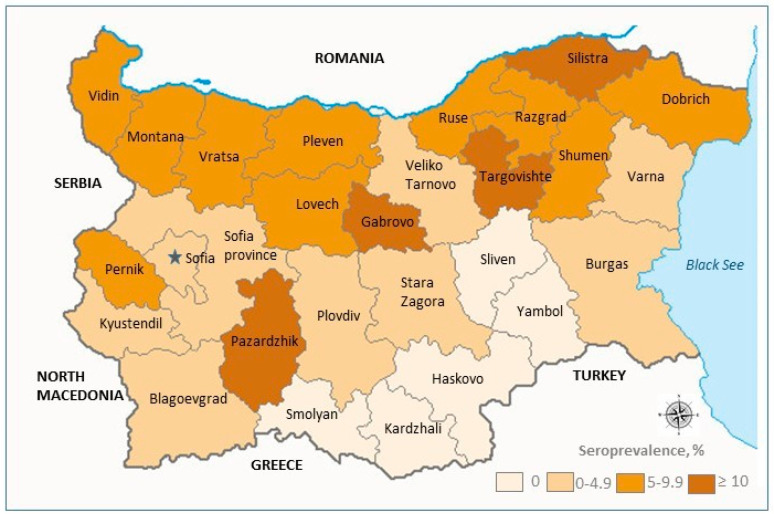
Geographic distribution of *Lyme borreliosis* seroprevalence in Bulgaria, 2023.

The seroprevalence of Lyme disease in the provinces ranged from 0.0% to 20.0%. No specific Anti-*Borrelia* IgG antibodies were detected in five provinces: Kardzhali, Sliven, Smolyan, Haskovo, and Yambol. Lower-to-medium seroprevalence levels, up to 5.0%, were observed in the central provinces and the provinces along the Black Sea coast. Higher seroprevalence rates were found in the northern part of the country, with the highest values in Gabrovo (18.9%) and Targovishte (20.0%). Seroprevalence data for each province are listed in Table 2 (in descending order) and depicted on the map in Figure 2. Overall, the seroprevalence in the northern part of the country is nearly four times higher than in the south, with mean values of 8.9% and 2.3%, respectively. 

## 4. Discussion

To our knowledge, this is the first nationwide study on the prevalence of *Lyme borreliosis* in Bulgaria. We found an overall LB seroprevalence of 5.4% (102/1892) among the general population. A previous small study conducted in 2015–2017 in Pleven province observed an IgG seropositivity of 6.32% among individuals with a history of a tick bite [27]. This finding is consistent with our current results for the province, which show a seropositivity rate of 6.7%. Few studies on the seroprevalence of LB have been published in other countries in the region, most of which at a subnational level. There is considerable variation in the testing strategies employed across these studies, including differences in the number and type of test methods used and the type of antibodies tested (IgG and/or IgM). This makes the reported results difficult to compare due to the different sensitivity and specificity of the testing protocols used.

A study from Romania conducted among healthy blood donors from six counties reported that 2.3% (28/1200) of the tested samples were positive and 1.75% (21/1200) were equivocal for IgG antibodies against *Bbsl* [20]. A study from Serbia found a seropositivity of 5.7% (2/35) among healthy blood donors from the Belgrade area [21]. However, a study from Novi Sad, Serbia, and Skopje, North Macedonia, found that seropositivity rates among healthy donors were 16.12% (10/62) and 2.17% (1/46), respectively, but in this case, serum samples were tested only by the ELISA method [28].

In Turkey, studies on the seroprevalence of LB have been conducted mainly in provinces in the western part of the country, which are located at a far distance from Bulgaria. Overall, when considering only the studies using the two-tier approach, the reported seropositivity rates among the general population range from 0.0% to 14.5% in the different provinces [22,23,24,25,26,29]. In contrast, in Greece, to date, only one seroprevalence study among healthy individuals is available, conducted in 2000, in which 0.27% (3/1100) of the tested samples were IgG-positive by Western blot [30].

In our study, we identified an increased risk of seropositivity associated with sex, age, and rural residence. The highest seroprevalence rates were found in adults over 65 years. This observation aligns with findings from studies conducted in Finland, France, Turkey, Germany, Austria, Belgium, and Poland [3,31,32]. Some authors attribute the higher seropositivity rates in older adults to factors such as increased outdoor activities, biological and immunological characteristics [33], and cumulative exposure to tick bites [34]. Additionally, the prevalence of specific antibodies was more than twice as high in males compared to females and in participants living in rural areas compared to those in urban areas. The higher risk in males is typically linked to more frequent exposure to tick bites due to occupational and recreational activities [35]. Living in rural areas may increase the risk of tick bites due to proximity to tick habitats, higher wildlife populations, and engagement in outdoor activities like farming, gardening, and hiking [36].

Regarding the prevalence of LB across different regions of Bulgaria, seroprevalence rates among all 28 provinces range from 0.0% to 20.0%. Lower rates (up to 5%) or the absence of specific anti-*Borrelia* antibodies were observed in the southern and eastern parts of the country. An exception is Pazardzhik province, located in central southern Bulgaria, where the seroprevalence was 10.0%. Despite this, the incidence of *Lyme borreliosis* in Pazardzhik is relatively low [7], and the area is not considered highly endemic for the disease. The available epidemiological data in this study are limited (ex. occupation or outdoor activities of the participants), and further investigations are necessary to verify the consistency of these results. Bulgaria has several main climatic zones from north to south: temperate continental, transitional continental, transitional Mediterranean, and a distinct zone in the eastern coastal area. Our study found a pronounced association between the regional climatic features and *Lyme borreliosis* prevalence, with seroprevalence increasing from south to north as the climate becomes more continental. 

The limitations of this study are related to the duration of the specific IgG immune response, as well as the possibility that people who were treated early in the course of infection and never developed IgG antibodies might have been included. Furthermore, this approach cannot distinguish between active and past infection. The study population included persons over 18 years of age, and the prevalence among younger ages could not be assessed.

Finally, in our study, about 30% of borderline and positive ELISA results for IgG antibodies were confirmed by the immunoblot method. This highlights the importance of using two-step testing protocols in routine laboratory practice for diagnosing *Lyme borreliosis*.

Current data on *Lyme borreliosis* prevalence in Bulgaria are crucial and could enhance awareness among public health authorities, clinical specialists, general practitioners, laboratories, and the general population. This knowledge can help in understanding risks, considering preventive measures, and improving the diagnosis, treatment, and management of the infection.

## Figures and Tables

**Table 1:** *Lyme borreliosis* seropositivity (% of positive samples) in the Bulgarian population by age groups (18–39, 40–65, and over 65), sex, and place of residence.

		Tested Samples, n (%)	Positive Samples, n (%)	95% CI
**Age, years**				
	18–39	406 (20.5%)	11 (2.7%)	1.4–4.8
	40–65	868 (43.8%)	39 (4.5%)	3.2–6.1
	over 65	618 (31.2%)	52 (8.4%)	6.3–10.9
**Sex**				
	male	778 (41.1%)	65 (8.4%)	6.5–10.5
	female	1114 (58.9%)	37 (3.3%)	2.3–4.6
**Residence**				
	city	1578 (83.4%)	70 (4.4%)	3.5–5.6
	village	314 (16.6%)	32 (10.2%)	4.4–6.5
Total population		1892 (100%)	102 (5.4%)	4.4–6.5

**Table 2:** *Lyme borreliosis* seroprevalence in Bulgaria (2023).

Province	Tested Samples, n	Positive Samples, n	Seroprevalence, %	95% CI
Overall	1892	102	5.4	4.4–6.5
Kardzhali	65	0	0.0	-
Sliven	60	0	0.0	-
Smolyan	60	0	0.0	-
Haskovo	100	0	0.0	-
Yambol	60	0	0.0	-
Burgas	95	1	1.1	0.0–5.7
Sofia province	80	1	1.3	0.0–6.8
Plovdiv	70	1	1.4	0.0–7.7
Blagoevgrad	63	1	1.6	0.0–8.5
Sofia city	60	1	1.7	0.0–8.9
Kyustendil	50	1	2.0	0.1–10.7
Varna	60	2	3.3	0.4–11.5
Stara Zagora	60	2	3.3	0.4–11.5
Veliko Tarnovo	64	3	4.7	1.0–13.1
Dobrich	60	3	5.0	1.0–13.9
Ruse	60	3	5.0	1.0–13.9
Pernik	70	4	5.7	1.6–14.0
Shumen	82	5	6.1	2.0–13.7
Lovech	60	4	6.7	2.0–16.2
Montana	60	4	6.7	1.9–16.2
Pleven	60	4	6.7	1.9–16.2
Vratsa	58	4	6.9	1.9–16.7
Vidin	40	3	7.5	1.6–20.4
Razgrad	60	5	8.3	2.8–18.4
Pazardzhik	100	10	10.0	4.9–17.6
Silistra	60	6	10.0	3.8–20.5
Gabrovo	95	18	18.9	11.6–28.3
Targovishte	80	16	20.0	11.9–30.4

CI = confidence interval.

## Data Availability

Data are contained within the article.

## Supplementary Materials

The following are available online at https://www.mdpi.com/article/10.3390/pathogens13090754/s1, Table S1: Logistic regression analysis of the Lyme seropositivity over the age, sex, and place of residence of the participants.
