# Supplementary material for: Serological Assessment of Lyme borreliosis in Bulgaria: A Nationwide Study
Source: Pathogens. 2024 Sep 2;13(9):754. doi: 10.3390/pathogens13090754 (PMC11435333; doi:10.3390/pathogens13090754)
Supplement: Supplementary file 1 [file pathogens-13-00754-s001.zip › pathogens-3168569-supplementary.pdf]

Model		Unstandardized coefficients		Standardized Coefficients	t	Significance	95.0% Confidence Interval for B	
		B	Standard error	Beta			Lower Bound	Upper Bound
	(Constant)	2.011	.030		68.060	.000	1.953	2.069
	Sex	.043	.011	.094	4.087	.000	.022	.064
	Place of residence	-.051	.014	-.084	-3.696	.000	-.078	-.024
	Age	-.001	.000	-.103	-4.464	.000	-.002	-.001
Dependent Variable: seropositivity								

Table S1. Logistic regression analysis of the Lyme seropositivity over the age, sex and place of residence of the participants.
